# Feasibility of the area reduction post-closure technique for bedside weaning of veno-arterial extracorporeal membrane oxygenation

**DOI:** 10.3389/fcvm.2024.1522789

**Published:** 2025-01-15

**Authors:** Chen Xu, Guo-xiong Xu, Yi-fei Cao, Lei Chen, Yi-qi Jin

**Affiliations:** ^1^ Department of Vascular and Endovascular Surgery, The Affiliated Suzhou Hospital of Nanjing Medical University, Suzhou, China; ^2^ Department of Critical Care Medicine, The Affiliated Suzhou Hospital of Nanjing Medical University, Suzhou, China

**Keywords:** the area reduction post-closure technique, bedside weaning, veno-arterial extracorporeal membrane oxygenation, ProGlide devices, access bleeding

## Abstract

**Objective:**

To evaluate the safety and efficacy of the area reduction post-closure technique for bedside weaning of veno-arterial extracorporeal membrane oxygenation (V-A ECMO).

**Methods:**

A retrospective study was conducted from December 2022 to November 2023, analyzing data from patients who underwent V-A ECMO weaning at our center. The area reduction post-closure technique, utilizing two ProGlide devices (Abbott Vascular, Santa Clara, CA), was adopted as a standard practice. The technical success was defined as achieving complete hemostasis without a bailout open repair. The complications associated with access included hemorrhagic events, pseudoaneurysm formation, limb ischemia, distal embolization, and wound infections.

**Results:**

A total of 18 patients were included. The median age of the cohort was 72.0 years [interquartile range (IQR), 57.5–81.5 years], with a male-to-female ratio of 2:1. The median size of arterial sheath utilized was 18.0 Fr (IQR, 17.0–20.0 Fr). The median duration of the procedure was 10.0 min (IQR, 9.0–13.0 min), and the median length of total hospital stay was 31.0 days (IQR, 25.5–39.0 days). Furthermore, the technique demonstrated a success rate of 100%. One patient (5.6%) experienced minor bleeding, which was successfully managed through compression. No additional complications associated with access were observed after the procedure.

**Conclusions:**

The post-closure area reduction technique emerges as a viable option for bedside weaning of V-A ECMO. Nonetheless, it is essential that this technique be validated through larger comparative studies.

## Introduction

The global adoption of percutaneous mechanical circulatory support (MCS) devices has experienced a consistent upward trend over the past decade (1). Veno-arterial extracorporeal membrane oxygenation (V-A ECMO) is primarily utilized for patients exhibiting unstable hemodynamics and significant gas exchange impairments, and it can be rapidly implemented in emergency situations (2–4). However, the large arterial cannula size has raised concerns regarding access-related complications after V-A ECMO decannulation (5, 6). Compared with the open repair, the percutaneous removal technique utilizing Proglide devices (Abbott Vascular, Santa Clara, California, USA) has demonstrated improved patient comfort and a reduced procedure duration (7).

The pre-closure technique has been widely reported for weaning process of V-A ECMO (8, 9). However, this technique can prolong the time needed for the insertion of V-A ECMO. The presence of indwelling sutures may increase the risk of wound infections and vascular injury, while also enhancing the care requirement. Recent studies have investigated the feasibility of post-closure techniques during V-A ECMO decannulation (10–12). However, the existing data remains limited, and the challenges related to effectively capturing vascular wall tissue within the large arterial cannula hole are concerning. Therefore, this study aimed to evaluate the safety and efficacy of the area reduction post-closure technique for bedside weaning of V-A ECMO.

## Methods

### Study population

**Figure 1:**
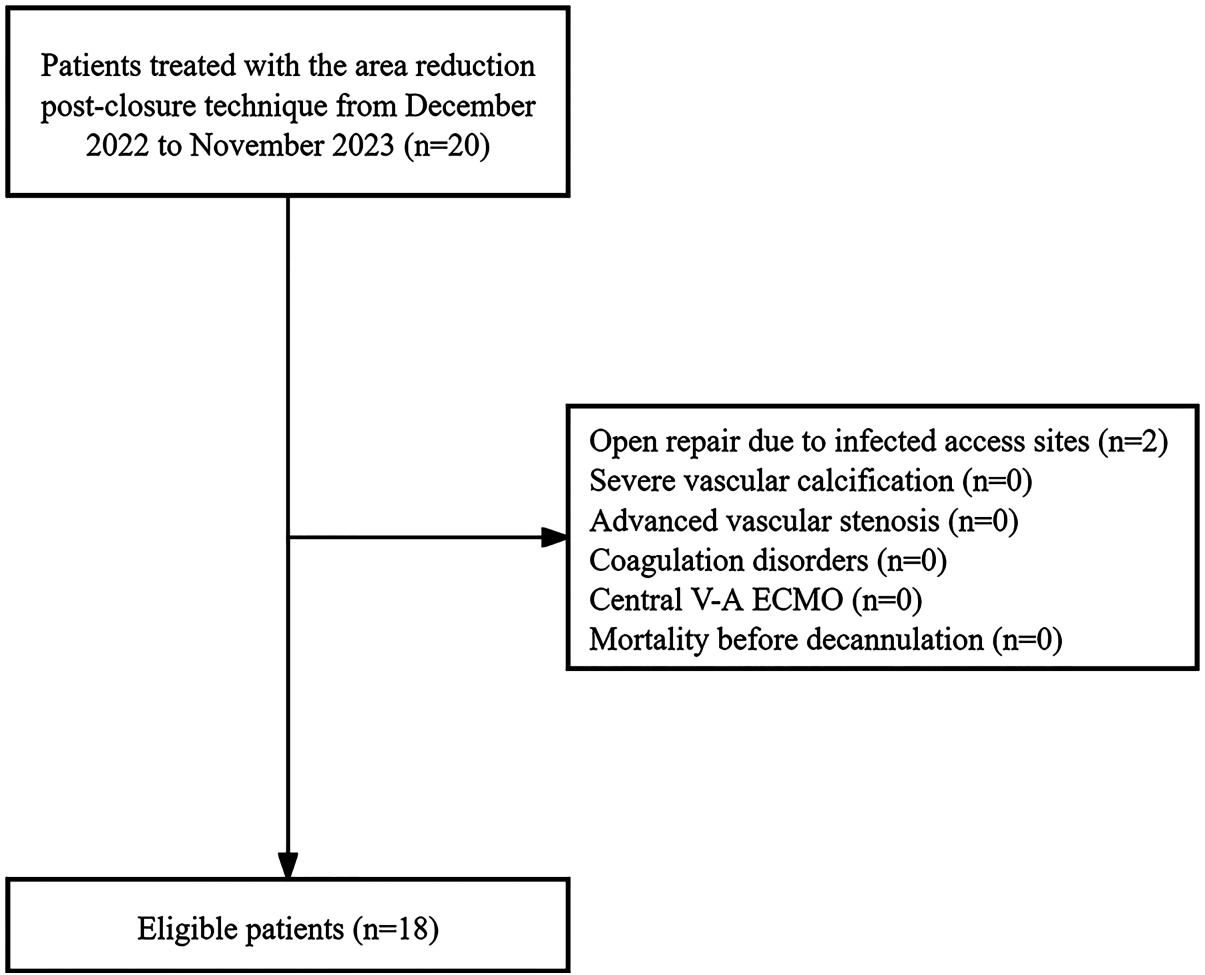
The study flowchart.

This study involved all patients who underwent bedside weaning of V-A ECMO at our center from December 2022 to November 2023. Patients treated with the area reduction post-closure technique were included. Exclusion criteria included severe vascular calcification, advanced vascular stenosis, coagulation disorders, the need for an open repair approach due to active bleeding or infected access sites, central V-A ECMO, and mortality before decannulation (Figure 1). V-A ECMO was utilized in cases where patients in shock did not demonstrate a favorable response to vasopressor therapy, coupled with the acute cardiopulmonary failure. The institutional committee approved this study including any relevant details, and the informed consent requirement was waived due to the retrospective and anonymous nature of the analysis.

### Technique details

**Figure 2:**
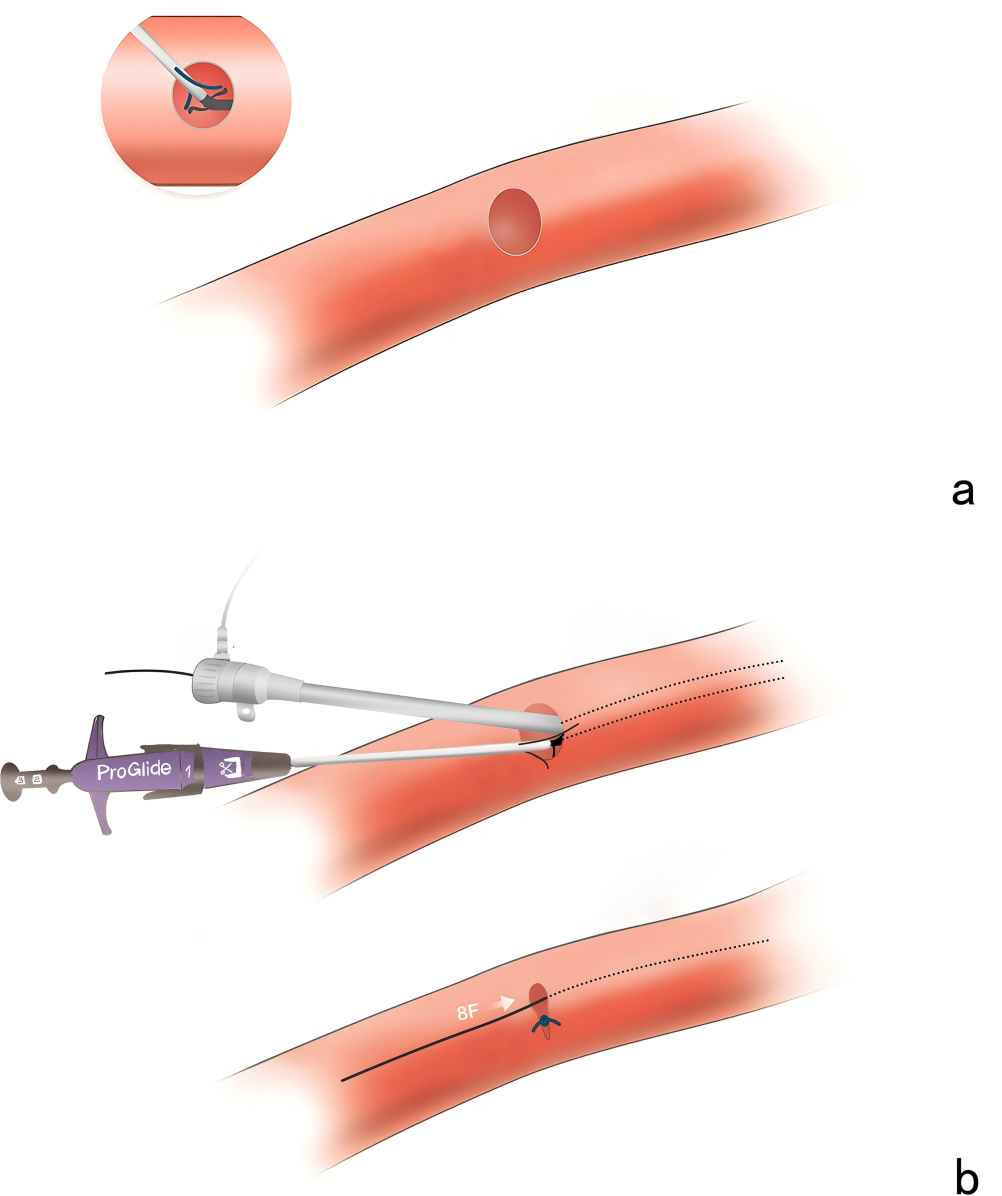
The area reduction post-closure technique. **(a)** The inability to capture a sufficient portion of the vascular wall tissue to allow adequate sealing. **(b)** The large arterial cannula hole was shrunk to 8 Fr in diameter after suturing the first Proglide device.

The area reduction post-closure technique involves a double-wire approach to deploy two Proglide devices gradually reducing the area of the large arterial cannula hole during weaning process of V-A ECMO at the patient's bedside, therefore achieving complete hemostasis (Figure 2). All procedures were performed by the same team. Two operators were recommended to finish the procedure at least: one performing the percutaneous closure, another performing bedside ultrasound and controlling bleeding. Notably, a large sheath with size up to 12–16 Fr was prepared for standby insertion to control the bleeding in case of closure failure. During the procedure, it is essential to incorporate volume management strategies alongside the evaluation of the patient's circulatory status and tissue perfusion. It is advisable to meticulously regulate fluid intake, promptly manage any instances of volume overload, sustain a relatively low volume status, diminish both preload and post-load on the heart, reduce venous pressure, improve organ perfusion, and utilize continuous renal replacement therapy when indicated. The administration of heparin was discontinued 30–60 min before decannulation, and a brief monitoring period was required to confirm stable hemodynamic conditions.

**Figure 3:**
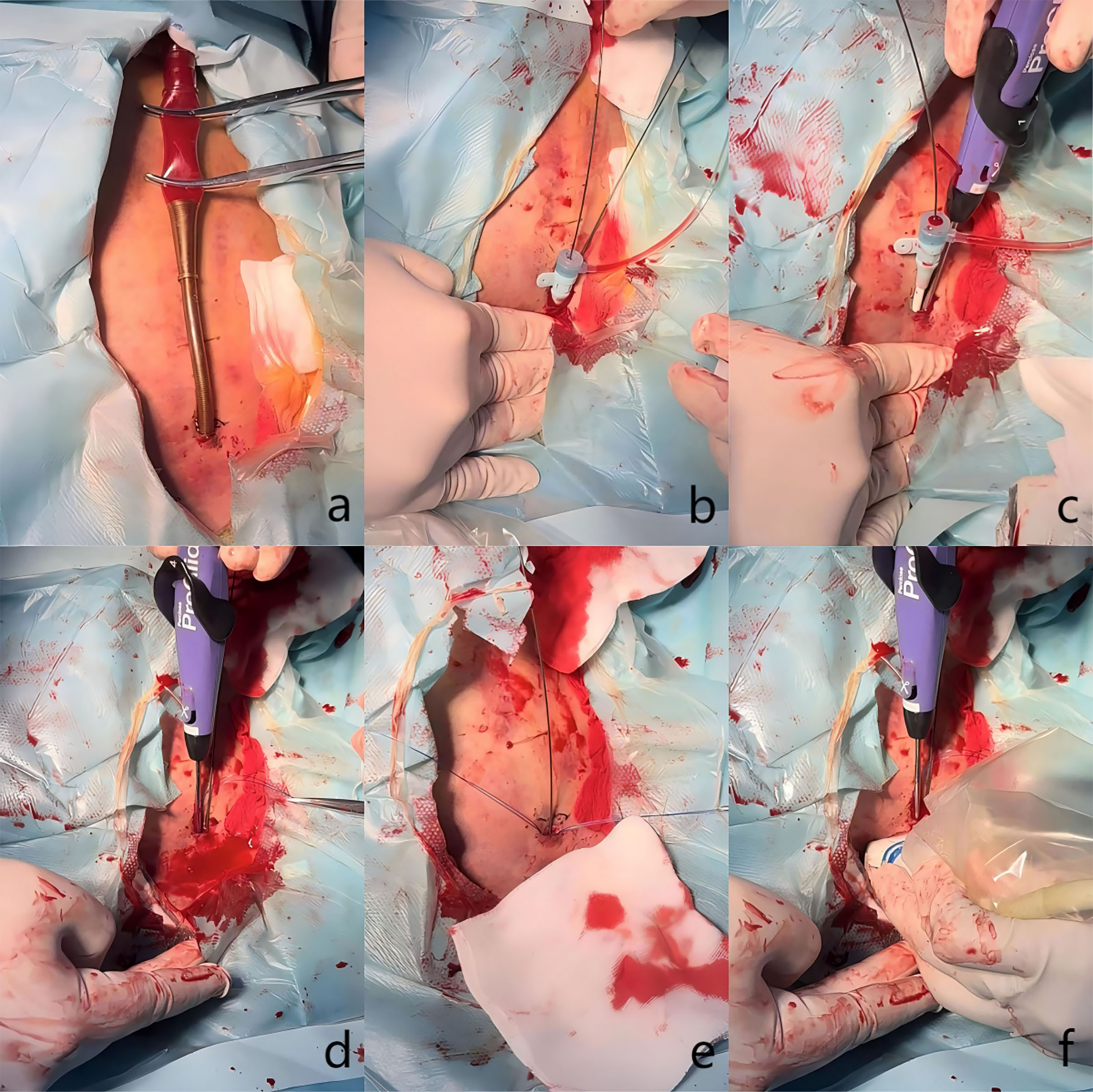
Clinical details of the technique. **(a)** The arterial cannula was clamped with two forceps. **(b)** Two guidewires were advanced and an 8 Fr sheath was introduced. **(c)** The first Proglide device was deployed to control bleeding. **(d)** The second Proglide device was deployed to control bleeding. **(e)** Hemostasis was achieved. **(f)** Proper position of the device's foot pedal was confirmed through ultrasound examination.

**Figure 4:**
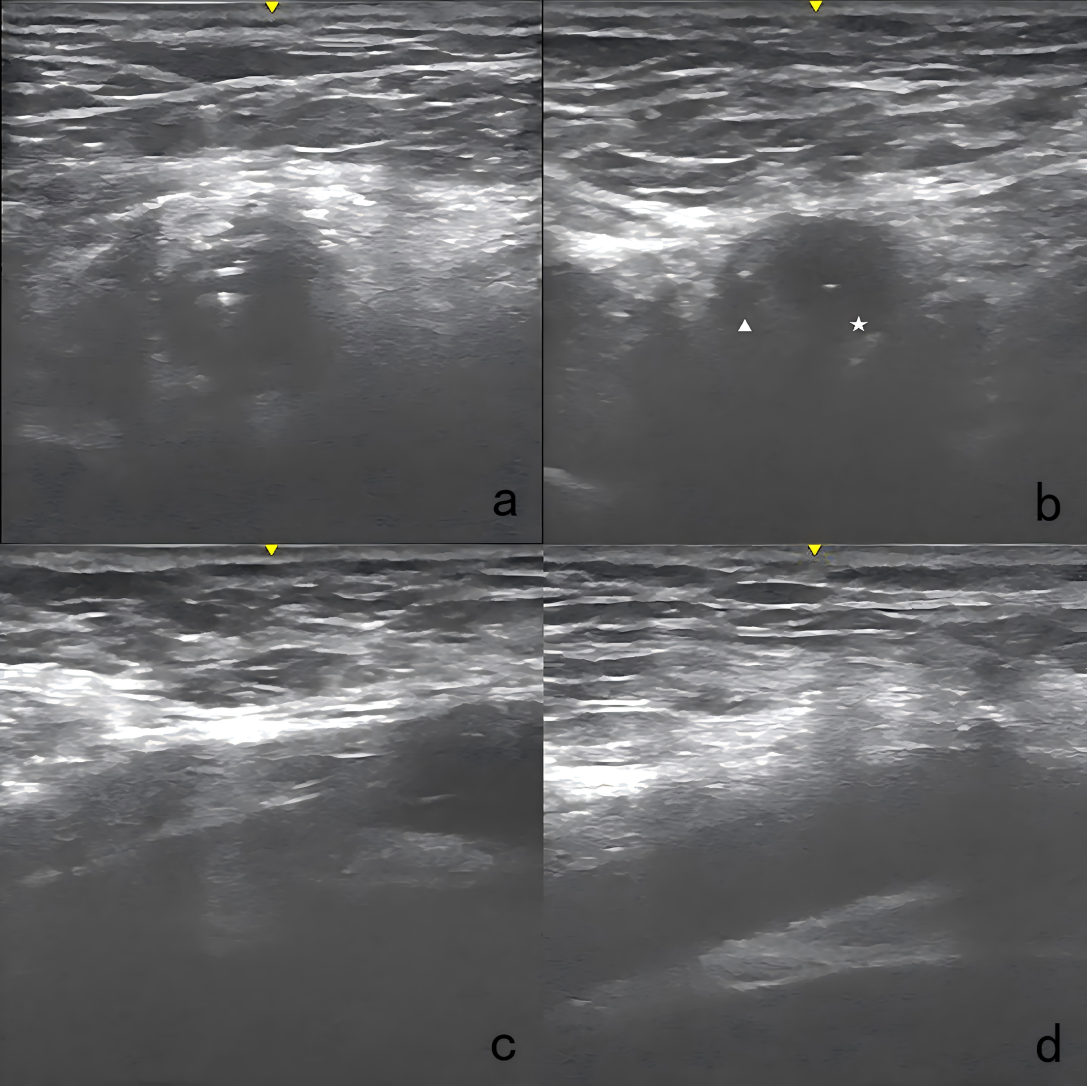
Ultrasound images of the technique. **(a)** Two guidewires were advanced in the arterial cannula. **(b)** An 8 Fr sheath was introduced over the first guide wire, positioned parallel to the second guidewire. Pentacle: the 8 Fr sheath; triangle: the remaining vascular lumen. **(c)** The foot pedal of the Proglide device was confirmed to be abutting the vessel wall. **(d)** The access site was examined right after the procedure with ultrasound to detect any potential complications.

Figure 3 illustrates the details of the technique. After disinfection, the arterial cannula was clamped with two forceps (Maquet, Germany, 20 cm), and the V-A ECMO flow was halted as expected. A direct puncture was performed in the proximal section of the arterial cannula with the Seldinger technique. The first guidewire (0.035-inch, Terumo, Tokyo, Japan) was inserted, and an 8 Fr sheath (Terumo, Somerset, NJ) was introduced. The second guidewire was inserted through the 8 Fr sheath. While one operator applied manual compression to the access, the 8 Fr sheath and V-A ECMO arterial cannula were carefully removed, ensuring the guidewire remained in position. The 8 Fr sheath was reintroduced over the first guidewire. The first Proglide device was deployed over the second guidewire at a 10 o'clock angle, and the suturing was carried out to control bleeding. Subsequently, the large arterial cannula hole was shrunk to 8 Fr in diameter. After removing the sheath, the second Proglide device was deployed over the first guidewire at a 2 o'clock angle to achieve further hemostasis. The first guidewire remained in place until complete hemostasis was conformed. If significant bleeding occurs, a third Proglide device will be deployed at a 12 o'clock angle over the safety guidewire. If hemostasis is not achieved after that, the procedure is considered unsuccessful, and a large sheath should be inserted to control bleeding. Emergency open repair should be arranged quickly in the operating room. Notably, an ultrasound examination was utilized at the operator's discretion during the deployment of Proglide device to confirm that the foot pedal are correctly positioned against the vessel wall rather than the adjacent soft tissue (Figure 4).

Finally, the manual compression was performed for several minutes. All access were examined right after the procedure and again 24 h later, utilizing ultrasound to detect any access-related complications.

### Definitions and follow-up

The primary outcome was the technical success, which was defined as achieving complete hemostasis without the need for an emergency open repair. The secondary outcome was the access-related complications, including hemorrhagic events, pseudoaneurysm formation, limb ischemia, distal embolization, and wound infections. Minor bleeding encompassed skin bruising and hematomas. Major bleeding was considered as a significant drop in hemoglobin levels that required a blood transfusion. Furthermore, the additional ues of Proglide device, the duration of the procedure, the duration of V-A ECMO support, the duration of intensive care unit (ICU) stay after weaning, the length of total hospital stay, the need for V-A ECMO reinsertion, and in-hospital mortality were recorded and analyzed. The follow-up ultrasound was utilized before discharge to evaluate any complications related to the access. Detailed clinical data were obtained from the online clinical and standard operative records.

### Statistical analysis

The distributed data is presented as the interquartile range (IQR). Categorical data is represented as counts and percentages. Statistical analyses were conducted using SPSS software (version 19.0; SPSS Inc., Chicago, IL, USA).

## Results

### Baseline characteristics

Two patients required open repair for V-A ECMO decannulation due to infected access sites. Subsequently, 18 patients utilizing the area reduction post-closure technique were included. Table 1 presents the baseline characteristics. The median age of the cohort was 72.0 years (IQR, 57.5–81.5 years), with a male-to-female ratio of 2:1. The median size of arterial sheath utilized was 18.0 Fr (IQR, 17.0–20.0 Fr).

**Table 1:** Baseline characteristics (*n* = 18).

Age, years	72.0 (57.5, 81.5)
Sex, male	12 (66.7)
Body mass index, kg/m^2^	23.9 (20.9, 29.3)
Comorbidities	
Hypertension	10 (55.6)
Diabetes mellitus	8 (44.4)
Coronary artery disease	8 (44.4)
Chronic kidney disease	4 (22.2)
Current smoking	5 (27.8)
Indication	
Cardiogenic failure	15 (83.3)
Respiratory failure	2 (11.1)
Septic shock	1 (5.6)
Arterial cannula size, Fr	
16	2 (11.1)
17	6 (33.3)
18	4 (22.2)
20	6 (33.3)
Median size of arterial sheathe, Fr	18.0 (17.0, 20.0)
Ipsilateral venous cannula	16 (88.9)
Cardiopulmonary resuscitation at insertion	10 (55.6)
Continuous renal replacement therapy	4 (22.2)
Anticoagulant treatment	18 (100.0)
Antiplatelet treatment	
SAPT	3 (16.7)
DAPT	8 (44.4)
Previous open repair or vascular closure device use	8 (44.4)

INR, International normalized ratio; SAPT, single antiplatelet therapy; DAPT, dual antiplatelet therapy.

Values are median with interquartile ranges (IQR) or number (%).

### Clinical outcomes

Table 2 presents the clinical outcomes. The technical success rate was 100%. Two patients (11.1%) accepted additional Proglide deployment, with one device utilized for each patients. One patient experienced (5.6%) minor bleeding that was successfully managed with manual compression, and there were no other access-related complications during the study period. The total access-related complication rate was 5.6%. Furthermore, the median duration of the procedure was 10.0 min (IQR, 9.0–13.0 min). No instances of V-A ECMO reinsertion were observed, and the in-hospital mortality rate was recorded at 16.7%.

**Table 2:** Clinical outcomes (*n* = 18).

Technical success	100%
Additional use of Proglide device	2 (11.1)
Access-related complications	
Minor bleeding	1 (5.6)
Major bleeding	0
Other	0
Duration of procedure, min	10.0 (9.0, 13.0)
Duration of V-A ECMO support, days	7.0 (6.0, 9.5)
Duration of ICU stay after weaning, days	15.0 (13.0, 19.5)
Length of hospital stay, days	31.0 (25.5, 39.0)
Need for ECMO reinsertion	0
In-hospital mortality	3 (16.7)

V-A ECMO, veno-arterial extracorporeal membrane oxygenation; ICU, intensive care unit.

Values are median with interquartile ranges (IQR) or number (%).

## Discussion

This single-center study evaluated the safety and efficacy of the area reduction post-closure technique for bedside weaning of V-A ECMO. The initial results indicated that the technical success rate was 100%, and the total access-related complication rate was 5.6%. Overall, this technique was a feasible and safe strategy and may be considered as a viable option.

The Proglide-assisted pre-closure technique has gained considerable attention and application for weaning process of V-A ECMO (8, 9). However, this technique may not be applicable to all cases of V-A ECMO, particularly in urgent situations such as cardiac arrest or cardiogenic shock, where the timely insertion is critical. Furthermore, the large arterial cannula system is typically not removed immediately, which may elevate the risk of wound infections and vascular injury, while also increase the care requirement. Recently, Hwang et al. (7) have reported a standard post-closure technique utilizing two Proglide devices. Their findings indicate that post-closure removal is not inferior to surgical removal in terms of the procedural outcomes and complications. A significant distinction between their technique and the area reduction technique lies in the size of the cannula utilized. The present study predominantly utilizes the 20 Fr cannulas. Theoretically, there is a concern of the capacity to obtain an adequate segment of the vascular wall tissue directly through a single Proglide in a large arterial cannula hole, which may hinder effective sealing. Hayakawa et al. (10) have described a post-closure technique that involves access to the contralateral femoral artery. This technique requires inserting and inflating a balloon while deploying two Proglide devices to achieve hemostasis. However, the contralateral femoral access and additional balloon insertion may increase the technical complexity.

This study presents the area reduction post-closure technique for the bedside weaning of V-A ECMO. The area was significantly reduced through the advancement of an 8 Fr sheath, which facilitated the effective deployment of the first Proglide device. Subsequently, the closure of the remaining 8 Fr hole can be achieved through the second Proglide device. Notably, the potential applicability of this technique to larger cannula sizes would further evaluate its advantages in comparison to the standard post-closure method. Furthermore, the access-related complication rate was 5.6%, aligning with conventional pre-closure studies that reported ranging from 4.8% to 28.6% (8, 9, 13).

In this study, an 8 Fr sheath was routinely utilized to reduce the cross-sectional area of the large cannula. However, it is conceivable that the application of an 8 Fr sheath may not completely preclude the placement of a ProGlide device at the center. In cases where larger cannula sizes are employed, such as a 24 Fr cannula, a sheath size ranging from 10 to 12 Fr may be required. Further studies is warranted to determine whether the cannula size for ECMO affects the appropriate sheath size. Additionally, the lack of fluoroscopy can be considered as a disadvantage that affects the technical reliability. However, there are advantages to weaning patients from V-A ECMO at the bedside. Firstly, it reduces the risk of complications that can arise during the transport of critically ill patients, which often involves moving a large amount of equipment and infusion lines, making the process more complex. Secondly, it can be performed at any time without the need for prior coordination with surgical team, allowing for a more prompt procedure for the patient. Further studies are need to evaluate the safety and efficacy of beside decannulation compared to that performed in the operating room under fluoroscopy.

This study is subject to several limitations, notably its relatively small sample size and its single-center design. It is important to highlight that the lack of a comparison group may affect the reliability of the results. Furthermore, patients deemed suitable for an open repair, such as those requiring infection debridement, hemostasis, or embolectomy were excluded from the study. This exclusion effectively restricts the study population to patients with lower risk profiles, where the effectiveness of percutaneous vascular closure devices (VCDs) is expected to be greater. Overall, there is a need for larger prospective studies that include an appropriate comparison group.

## Conclusion

The area reduction post-closure technique was a feasible and safe strategy for bedside weaning of V-A ECMO, and may be considered as a viable option.

## Data Availability

The raw data supporting the conclusions of this article will be made available by the authors, without undue reservation.
