# Supplementary material for: Feasibility of the area reduction post-closure technique for bedside weaning of veno-arterial extracorporeal membrane oxygenation
Source: Front Cardiovasc Med. 2025 Jan 15;11:1522789. doi: 10.3389/fcvm.2024.1522789 (PMC11774842; doi:10.3389/fcvm.2024.1522789)
Supplement: Supplementary file 2 [file Image1.pdf]

Feasibility of the Area Reduction Post-closure Technique for Bedside Weaning of Veno-arterial Extracorporeal Membrane Oxygenation

Eighteen patients ubdergoing

the area reduction post-closure technique

included in this study

↓

Median age was 72.0 years

Male-to-female ratio was 2:1

Median size of arterial sheath was 18.0 Fr

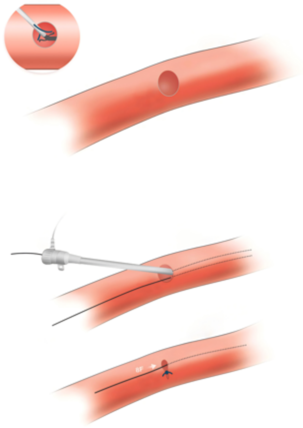

Clinical outcomes	
Technique success	100%
Additional use of Proglide device	2 (11.1)
Access-related complications	
Minor bleeding	1 (5.6)
Major bleeding	0
Pseudoaneurysm formation	0
Limb ischemia	0
Distal embolization	0
Wound infection	0
Duration of procedure, min	10.0 (9.0, 13.0)
Duration of V-A ECMO support, days	7.0 (6.0, 9.5)
Duration of ICU stay after weaning, days	15.0 (13.0, 19.5)
Length of thospital stay, days	31.0 (25.5, 39.0)
Need for ECMO reinsertion	0
In-hospital mortality	3 (16.7)

Conclusion: The area reduction post-closure technique was a feasible and safe strategy for VA-ECMO bedside weaning and may be considered an alternative option.
